# Supplementary material for: Evaluating Effectiveness of Public Health Intervention Strategies for Mitigating COVID-19 Pandemic
Source: ArXiv. 2021 Jul 20:arXiv:2107.09749v1. Preprint. [Version 1] (PMC8312897)
Supplement: 1 [file NIHPP2107.09749V1-supplement-1.pdf]

Supporting information

Web Appendix A: R_t and Propensity Score Estimation

This section shows the results of estimated effective reproduction number R_t in the US (Figure S1), and the propensity score models for each intervention (Tables S1, S2, S3, S4, S5, and S6).

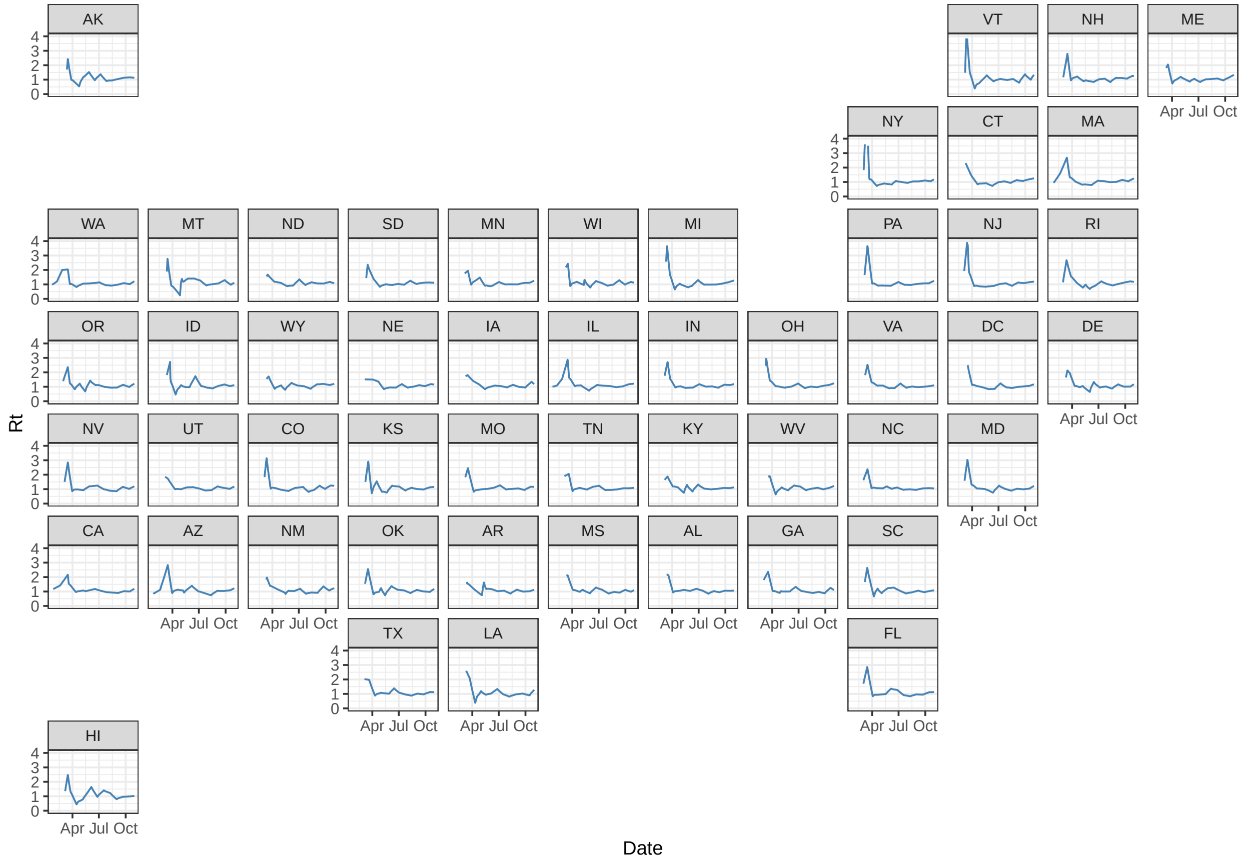

Figure S1: Estimated Effective Reproduction Number R_t From February 2020 to February 2021 in the US

Web Appendix B: HTE Results

This section shows the estimated HTE of race (percentage of White) for reopening bars (Figure S2). The effects are estimated with moderator fixed at a given quantile (e.g., 25th

Table S1: **Propensity Score Estimates of Lockdown**

Day	New Case $\widehat{\beta}$ (p-value)	R_t $\widehat{\beta}$ (p-value)	New Death $\widehat{\beta}$ (p-value)	Limit English $\widehat{\beta}$ (p-value)	Latino $\widehat{\beta}$ (p-value)	Multi-Unit House $\widehat{\beta}$ (p-value)	Institutionalized $\widehat{\beta}$ (p-value)	Crowded Household $\widehat{\beta}$ (p-value)
$\Delta = 1$	8.144 (0)	3.982 (0)	-64.394 (0.023)	-1.172 (0.001)	0.183 (0.001)	-0.068 (0.706)	1.189 (0.002)	0.953 (0.242)
$\Delta = 2$	11.901 (0)	5.085 (0)	-83.003 (0)	-1.493 (0.001)	0.220 (0)	-0.114 (0.599)	1.612 (0.003)	1.824 (0.120)
$\Delta = 3$	15.526 (0)	6.863 (0)	-116.992 (0)	-1.739 (0.007)	0.247 (0.003)	-0.095 (0.794)	2.242 (0.001)	2.320 (0.063)
$\Delta = 4$	28.865 (0)	8.230 (0)	31.493 (0.676)	-2.276 (0.002)	0.297 (0.003)	0.140 (0.632)	2.615 (0.018)	1.968 (0.203)
$\Delta = 5$	39.656 (0)	12.747 (0)	-56.959 (0.407)	-2.846 (0.005)	0.365 (0.015)	0.291 (0.279)	3.300 (0.003)	2.412 (0.015)
$\Delta = 6$	219.911 (0.107)	68.378 (0.09)	-558.381 (0.003)	-31.124 (0.104)	4.393 (0.102)	5.518 (0.062)	21.468 (0.153)	24.418 (0.226)
$\Delta = 7$	-	-	-	-	-	-	-	-
$\Delta = 8$	-	-	-	-	-	-	-	-
$\Delta = 9$	-	-	-	-	-	-	-	-
$\Delta = 10$	-	-	-	-	-	-	-	-
$\Delta = 11$	-	-	-	-	-	-	-	-
$\Delta = 12$	-	-	-	-	-	-	-	-
$\Delta = 13$	-	-	-	-	-	-	-	-
$\Delta = 14$	-	-	-	-	-	-	-	-

– indicates the variable was not applicable at Δ day.

Table S2: **Propensity Score Estimates of Stay-at-home**

Day	New Death $\widehat{\beta}$ (p-value)	New Case $\widehat{\beta}$ (p-value)	Limit English $\widehat{\beta}$ (p-value)	Latino $\widehat{\beta}$ (p-value)	R_t $\widehat{\beta}$ (p-value)	Multi-Unit House $\widehat{\beta}$ (p-value)	No High School Diploma $\widehat{\beta}$ (p-value)	Crowded Household $\widehat{\beta}$ (p-value)	Unemployed $\widehat{\beta}$ (p-value)	Institutionalized $\widehat{\beta}$ (p-value)	Disabled $\widehat{\beta}$ (p-value)
$\Delta = 1$	25.416 (0.007)	0.838 (0)	0.346 (0.279)	0.003 (0.941)	-0.201 (0.548)	-0.319 (0.169)	-0.618 (0.003)	0.473 (0.316)	-	-	-
$\Delta = 2$	27.021 (0.013)	0.993 (0)	0.436 (0.221)	-0.002 (0.965)	-0.259 (0.464)	-0.401 (0.089)	-0.739 (0.001)	0.616 (0.183)	-	-	-
$\Delta = 3$	31.587 (0.013)	1.168 (0)	0.609 (0.195)	0.002 (0.976)	-0.477 (0.241)	-0.519 (0.102)	-0.899 (0.003)	-	0.052 (0.934)	-	-
$\Delta = 4$	40.988 (0.019)	1.398 (0)	0.621 (0.059)	-0.003 (0.941)	0.055 (0.884)	-0.563 (0.018)	-0.989 (0.002)	0.904 (0.079)	-	-	-
$\Delta = 5$	48.896 (0.030)	1.746 (0.001)	0.836 (0.007)	-0.028 (0.513)	-	-0.612 (0.011)	-1.046 (0.001)	0.794 (0.159)	-	-0.155 (0.752)	-
$\Delta = 6$	46.312 (0.102)	2.070 (0)	0.891 (0.002)	-0.039 (0.408)	-	-0.589 (0.013)	-1.112 (0.004)	0.883 (0.118)	-	-0.347 (0.466)	-
$\Delta = 7$	29.690 (0.368)	3.021 (0)	0.743 (0.002)	-0.019 (0.728)	-	-0.575 (0.011)	-1.073 (0.006)	0.969 (0.073)	-	-0.340 (0.443)	-
$\Delta = 8$	17.192 (0.597)	3.901 (0.001)	0.049 (0.911)	0.007 (0.918)	-	-0.281 (0.270)	-	1.996 (0.023)	-2.166 (0.001)	-0.194 (0.759)	-
$\Delta = 9$	39.736 (0.314)	4.366 (0)	0.264 (0.640)	-0.079 (0.401)	-	-0.490 (0.161)	-	2.247 (0.003)	-1.943 (0.002)	-1.098 (0.063)	-
$\Delta = 10$	42.246 (0.381)	6.320 (0)	0.184 (0.792)	0.009 (0.905)	-	-0.373 (0.228)	-	2.082 (0.009)	-2.168 (0.034)	-	0.350 (0.699)
$\Delta = 11$	52.587 (0.410)	7.453 (0)	0.204 (0.706)	0.053 (0.418)	-	-0.225 (0.487)	-	2.187 (0.048)	-2.736 (0.017)	-	0.700 (0.445)
$\Delta = 12$	63.706 (0.418)	10.495 (0.006)	-0.982 (0.351)	0.179 (0.217)	-	0.524 (0.400)	-	2.616 (0.321)	-1.929 (0.119)	-	1.158 (0.145)
$\Delta = 13$	-	-	-	-	-	-	-	-	-	-	-
$\Delta = 14$	-	-	-	-	-	-	-	-	-	-	-

– indicates the variable was not selected or not applicable at Δ day.

Table S3: **Propensity Score Estimates of Mandatory Facial Mask**

Day	New Case $\widehat{\beta}$ (p-value)	R_t $\widehat{\beta}$ (p-value)	New Death $\widehat{\beta}$ (p-value)	No Vehicle $\widehat{\beta}$ (p-value)	Limit English $\widehat{\beta}$ (p-value)	Latino $\widehat{\beta}$ (p-value)	Unemployed $\widehat{\beta}$ (p-value)	Mobile Home $\widehat{\beta}$ (p-value)	Male at Age 65 and over $\widehat{\beta}$ (p-value)	Male $\widehat{\beta}$ (p-value)	White $\widehat{\beta}$ (p-value)
$\Delta = 1$	0.037 (0.007)	-1.942 (0.037)	0.374 (0.507)	-0.252 (0.422)	0.030 (0.935)	-0.013 (0.874)	0.589 (0.027)	-0.291 (0.060)	0.105 (0.539)	-	-
$\Delta = 2$	0.036 (0.004)	-2.281 (0.008)	0.316 (0.540)	-0.218 (0.459)	-0.031 (0.931)	0.004 (0.964)	0.580 (0.067)	-0.298 (0.048)	-	-0.062 (0.876)	-
$\Delta = 3$	0.037 (0.008)	-2.213 (0.008)	0.434 (0.386)	-0.221 (0.433)	-0.047 (0.897)	0.009 (0.912)	0.566 (0.071)	-0.277 (0.064)	-	-0.069 (0.872)	-
$\Delta = 4$	0.035 (0.009)	-2.034 (0.012)	0.663 (0.216)	-0.216 (0.433)	-0.057 (0.875)	0.013 (0.867)	0.521 (0.094)	-0.252 (0.083)	-	-0.016 (0.971)	-
$\Delta = 5$	0.073 (0)	-2.365 (0.009)	0.276 (0.607)	-0.257 (0.379)	-0.071 (0.839)	0.015 (0.838)	0.493 (0.122)	-0.346 (0.040)	-	0.013 (0.977)	-
$\Delta = 6$	0.069 (0.001)	-1.269 (0.148)	0.927 (0.142)	-0.381 (0.328)	0.201 (0.609)	-0.017 (0.836)	0.104 (0.739)	-	0.490 (0.104)	-0.426 (0.464)	-
$\Delta = 7$	0.080 (0)	-2.087 (0.018)	0.421 (0.448)	-0.359 (0.275)	-0.147 (0.654)	0.025 (0.731)	0.631 (0.054)	-0.419 (0.028)	-	-0.026 (0.955)	-
$\Delta = 8$	0.080 (0)	-2.090 (0.014)	0.410 (0.454)	-0.347 (0.286)	-0.141 (0.673)	0.019 (0.814)	0.715 (0.061)	-0.426 (0.027)	-	0.118 (0.837)	-
$\Delta = 9$	0.097 (0)	-1.526 (0.076)	0.704 (0.224)	-0.467 (0.135)	0.379 (0.292)	-0.033 (0.692)	1.213 (0.051)	-	-	0.418 (0.583)	0.099 (0.036)
$\Delta = 10$	0.097 (0)	-1.498 (0.078)	0.692 (0.232)	-0.461 (0.138)	0.378 (0.298)	-0.033 (0.695)	1.225 (0.048)	-	-	0.427 (0.572)	0.099 (0.035)
$\Delta = 11$	0.108 (0)	-1.357 (0.091)	0.502 (0.376)	0.003 (0.994)	0.268 (0.468)	-0.004 (0.965)	0.988 (0.137)	-	-	0.408 (0.587)	0.088 (0.077)
$\Delta = 12$	0.108 (0)	-1.350 (0.090)	0.478 (0.400)	0.005 (0.991)	0.269 (0.469)	-0.004 (0.961)	0.983 (0.143)	-	-	0.395 (0.603)	0.088 (0.079)
$\Delta = 13$	0.111 (0)	-1.367 (0.088)	0.642 (0.314)	-0.122 (0.765)	0.294 (0.445)	-0.014 (0.878)	1.166 (0.095)	-	-	0.471 (0.524)	0.097 (0.064)
$\Delta = 14$	0.121 (0)	-1.402 (0.112)	0.531 (0.421)	-0.099 (0.809)	0.290 (0.454)	-0.012 (0.896)	1.175 (0.098)	-	-	0.454 (0.550)	0.098 (0.066)

– indicates the variable was not selected at Δ day.

Table S4: **Propensity Score Estimates of Reopening Business**

Day	$\boldsymbol{R_t}$ $\widehat{\beta}$ (p-value)	**Limit English** $\widehat{\beta}$ (p-value)	**Multi-Unit House** $\widehat{\beta}$ (p-value)	**Mobile Home** $\widehat{\beta}$ (p-value)	**Latino** $\widehat{\beta}$ (p-value)	**Per Capita Income** $\widehat{\beta}\times 10^{-4}$ (p-value)	**Disabled** $\widehat{\beta}$ (p-value)	**No Vehicle** $\widehat{\beta}$ (p-value)	**Male at Age 65 and over** $\widehat{\beta}$ (p-value)	**Unemployed** $\widehat{\beta}$ (p-value)
$\Delta = 1$	-4.428 (0.001)	-0.257 (0.546)	-0.094 (0.645)	0.270 (0.039)	0.038 (0.583)	-0.036 (0.942)	-0.162 (0.265)	0.043 (0.769)	0.024 (0.900)	-
$\Delta = 2$	-4.678 (0.001)	-0.290 (0.524)	-0.091 (0.674)	0.301 (0.022)	0.041 (0.561)	-0.044 (0.931)	-0.164 (0.254)	0.039 (0.791)	0.013 (0.945)	-
$\Delta = 3$	-5.382 (0)	-0.322 (0.493)	-0.110 (0.627)	0.344 (0.005)	0.042 (0.555)	-0.024 (0.963)	-0.191 (0.172)	0.060 (0.688)	0.055 (0.771)	-
$\Delta = 4$	-5.707 (0)	-0.329 (0.504)	-0.116 (0.632)	0.363 (0.004)	0.041 (0.567)	-0.028 (0.957)	-0.192 (0.175)	0.053 (0.727)	0.059 (0.754)	-
$\Delta = 5$	-5.635 (0)	-0.342 (0.525)	-0.122 (0.649)	0.381 (0.003)	0.036 (0.633)	-0.010 (0.985)	-0.187 (0.221)	0.060 (0.723)	0.128 (0.491)	-
$\Delta = 6$	-6.222 (0)	-0.409 (0.470)	-0.123 (0.663)	0.432 (0.003)	0.042 (0.577)	0.055 (0.923)	-0.206 (0.191)	0.071 (0.692)	0.146 (0.440)	-
$\Delta = 7$	-6.572 (0)	-0.475 (0.464)	-0.126 (0.693)	0.427 (0.005)	0.054 (0.524)	-0.073 (0.899)	-0.222 (0.168)	0.070 (0.707)	0.133 (0.496)	-
$\Delta = 8$	-8.161 (0)	-0.362 (0.641)	-0.325 (0.419)	0.612 (0)	0.065 (0.492)	0.514 (0.416)	-0.198 (0.218)	0.193 (0.337)	0.167 (0.471)	-0.641 (0.111)
$\Delta = 9$	-8.768 (0)	-0.443 (0.602)	-0.308 (0.473)	0.645 (0)	0.073 (0.479)	0.345 (0.478)	-0.207 (0.259)	0.181 (0.384)	0.119 (0.630)	-0.624 (0.131)
$\Delta = 10$	-8.853 (0)	-0.440 (0.613)	-0.314 (0.468)	0.679 (0)	0.070 (0.502)	0.345 (0.600)	-0.251 (0.165)	0.177 (0.402)	0.099 (0.697)	-0.640 (0.131)
$\Delta = 11$	-9.031 (0)	-0.456 (0.598)	-0.290 (0.502)	0.665 (0)	0.069 (0.501)	0.224 (0.735)	-0.253 (0.161)	0.145 (0.504)	0.080 (0.753)	-0.670 (0.118)
$\Delta = 12$	-8.949 (0)	-0.485 (0.578)	-0.271 (0.525)	0.676 (0)	0.071 (0.488)	0.174 (0.793)	-0.285 (0.122)	0.128 (0.552)	0.058 (0.819)	-0.656 (0.131)
$\Delta = 13$	-9.090 (0)	-0.496 (0.570)	-0.263 (0.541)	0.668 (0)	0.072 (0.484)	0.141 (0.838)	-0.304 (0.133)	0.108 (0.635)	0.060 (0.822)	-0.700 (0.136)
$\Delta = 14$	-9.278 (0)	-0.497 (0.560)	-0.255 (0.539)	0.668 (0)	0.068 (0.504)	0.070 (0.920)	-0.323 (0.115)	0.088 (0.700)	0.044 (0.871)	-0.688 (0.146)

– indicates the variable was not selected at Δ day.

Table S5: **Propensity Score Estimates of Reopening Restaurants**

Day	R_t $\widehat{\beta}$ (p-value)	**Per Capita Income** $\widehat{\beta}\times 10^{-4}$ (p-value)	**Multi-Unit House** $\widehat{\beta}$ (p-value)	**Limit English** $\widehat{\beta}$ (p-value)	**Mobile Home** $\widehat{\beta}$ (p-value)	**Latino** $\widehat{\beta}$ (p-value)	**Single Parent House** $\widehat{\beta}$ (p-value)	**No Vehicle** $\widehat{\beta}$ (p-value)	**Disabled** $\widehat{\beta}$ (p-value)	**Poverty** $\widehat{\beta}$ (p-value)
$\Delta = 1$	-2.094 (0.039)	-0.362 (0.681)	-0.054 (0.782)	-0.140 (0.613)	0.085 (0.495)	0.004 (0.923)	0.113 (0.886)	-0.034 (0.850)	-0.227 (0.272)	0.114 (0.573)
$\Delta = 2$	-2.176 (0.035)	-0.379 (0.664)	-0.053 (0.784)	-0.159 (0.568)	0.071 (0.567)	0.007 (0.875)	0.108 (0.891)	-0.045 (0.805)	-0.226 (0.281)	0.126 (0.528)
$\Delta = 3$	-2.190 (0.036)	-0.444 (0.616)	-0.048 (0.811)	-0.137 (0.629)	0.089 (0.470)	0.000 (0.998)	0.163 (0.839)	-0.053 (0.772)	-0.250 (0.250)	0.126 (0.535)
$\Delta = 4$	-2.251 (0.034)	-0.441 (0.621)	-0.050 (0.799)	-0.119 (0.676)	0.101 (0.395)	-0.004 (0.925)	0.208 (0.798)	-0.065 (0.726)	-0.256 (0.251)	0.138 (0.510)
$\Delta = 5$	-2.624 (0.033)	-0.451 (0.613)	-0.070 (0.716)	-0.135 (0.626)	0.124 (0.298)	-0.005 (0.905)	0.148 (0.864)	-0.063 (0.741)	-0.315 (0.174)	0.159 (0.463)
$\Delta = 6$	-2.740 (0.030)	-0.482 (0.597)	-0.057 (0.768)	-0.145 (0.606)	0.130 (0.283)	-0.005 (0.921)	0.231 (0.791)	-0.079 (0.686)	-0.318 (0.175)	0.159 (0.475)
$\Delta = 7$	-2.823 (0.028)	-0.539 (0.558)	-0.068 (0.727)	-0.154 (0.576)	0.134 (0.285)	-0.001 (0.974)	0.282 (0.753)	-0.074 (0.708)	-0.320 (0.158)	0.145 (0.512)
$\Delta = 8$	-2.873 (0.027)	-0.679 (0.473)	-0.066 (0.745)	-0.155 (0.582)	0.138 (0.266)	-0.002 (0.963)	0.331 (0.728)	-0.073 (0.714)	-0.333 (0.145)	0.133 (0.555)
$\Delta = 9$	-3.035 (0.029)	-0.781 (0.431)	-0.076 (0.720)	-0.163 (0.565)	0.143 (0.242)	-0.005 (0.908)	0.451 (0.661)	-0.071 (0.730)	-0.374 (0.097)	0.143 (0.532)
$\Delta = 10$	-3.565 (0.037)	-0.950 (0.352)	-0.083 (0.701)	-0.177 (0.542)	0.153 (0.188)	-0.003 (0.938)	0.504 (0.624)	-0.067 (0.742)	-0.410 (0.080)	0.128 (0.593)
$\Delta = 11$	-3.596 (0.034)	-0.965 (0.342)	-0.081 (0.711)	-0.183 (0.536)	0.153 (0.203)	-0.004 (0.920)	0.545 (0.600)	-0.071 (0.732)	-0.407 (0.075)	0.124 (0.600)
$\Delta = 12$	-3.786 (0.029)	-1.018 (0.330)	-0.077 (0.730)	-0.195 (0.516)	0.140 (0.250)	-0.004 (0.926)	0.602 (0.569)	-0.085 (0.687)	-0.406 (0.082)	0.125 (0.595)
$\Delta = 13$	-3.860 (0.027)	-1.018 (0.314)	-0.086 (0.713)	-0.190 (0.524)	0.131 (0.285)	-0.005 (0.901)	0.537 (0.640)	-0.091 (0.677)	-0.408 (0.105)	0.136 (0.572)
$\Delta = 14$	-4.028 (0.024)	-1.180 (0.287)	-0.099 (0.669)	-0.177 (0.563)	0.118 (0.337)	-0.010 (0.819)	0.700 (0.562)	-0.097 (0.659)	-0.432 (0.093)	0.140 (0.572)

Table S6: **Propensity Score Estimates of Reopening Bars**

Day	**New Case** $\widehat{\beta}$ (p-value)	**New Death** $\widehat{\beta}$ (p-value)	**Limit English** $\widehat{\beta}$ (p-value)	**Multi-Unit House** $\widehat{\beta}$ (p-value)	**Per Capita Income** $\widehat{\beta}\times 10^{-4}$ (p-value)	**Latino** $\widehat{\beta}$ (p-value)	**Mobile Home** $\widehat{\beta}$ (p-value)	**Institutionalized** $\widehat{\beta}$ (p-value)
$\Delta = 1$	-0.060 (0.216)	-0.631 (0.378)	-0.320 (0.517)	0.032 (0.922)	-0.330 (0.648)	0.032 (0.560)	-0.066 (0.708)	0.270 (0.530)
$\Delta = 2$	-0.062 (0.197)	-0.598 (0.398)	-0.339 (0.498)	0.033 (0.920)	-0.373 (0.607)	0.032 (0.549)	-0.076 (0.673)	0.270 (0.523)
$\Delta = 3$	-0.063 (0.194)	-0.602 (0.394)	-0.345 (0.486)	0.038 (0.907)	-0.442 (0.549)	0.033 (0.535)	-0.078 (0.667)	0.258 (0.541)
$\Delta = 4$	-0.061 (0.209)	-0.626 (0.372)	-0.321 (0.528)	0.028 (0.934)	-0.412 (0.582)	0.030 (0.583)	-0.072 (0.692)	0.254 (0.544)
$\Delta = 5$	-0.062 (0.198)	-0.629 (0.370)	-0.319 (0.542)	0.022 (0.948)	-0.423 (0.575)	0.028 (0.605)	-0.070 (0.701)	0.231 (0.578)
$\Delta = 6$	-0.065 (0.179)	-0.654 (0.346)	-0.307 (0.558)	0.007 (0.984)	-0.377 (0.617)	0.029 (0.601)	-0.073 (0.700)	0.298 (0.482)
$\Delta = 7$	-0.068 (0.150)	-0.632 (0.359)	-0.338 (0.532)	0.005 (0.990)	-0.367 (0.632)	0.033 (0.553)	-0.085 (0.653)	0.283 (0.499)
$\Delta = 8$	-0.069 (0.148)	-0.644 (0.353)	-0.365 (0.500)	0.012 (0.973)	-0.361 (0.640)	0.035 (0.524)	-0.081 (0.674)	0.294 (0.486)
$\Delta = 9$	-0.071 (0.149)	-0.674 (0.359)	-0.468 (0.403)	0.056 (0.873)	-0.437 (0.589)	0.044 (0.427)	-0.073 (0.715)	0.301 (0.500)
$\Delta = 10$	-0.072 (0.138)	-0.658 (0.371)	-0.475 (0.406)	0.058 (0.870)	-0.475 (0.559)	0.044 (0.423)	-0.075 (0.711)	0.305 (0.485)
$\Delta = 11$	-0.077 (0.117)	-0.662 (0.365)	-0.489 (0.399)	0.044 (0.901)	-0.495 (0.543)	0.045 (0.420)	-0.091 (0.660)	0.340 (0.455)
$\Delta = 12$	-0.076 (0.117)	-0.711 (0.331)	-0.495 (0.407)	0.037 (0.920)	-0.544 (0.515)	0.044 (0.444)	-0.093 (0.658)	0.313 (0.491)
$\Delta = 13$	-0.077 (0.113)	-0.718 (0.321)	-0.517 (0.379)	0.045 (0.900)	-0.562 (0.500)	0.047 (0.410)	-0.098 (0.641)	0.337 (0.447)
$\Delta = 14$	-0.079 (0.110)	-0.760 (0.311)	-0.567 (0.346)	0.049 (0.892)	-0.645 (0.447)	0.051 (0.376)	-0.119 (0.589)	0.284 (0.528)

percentile, 50th percentile, 75th percentile) over all states and other covariates fixed at the mean level.

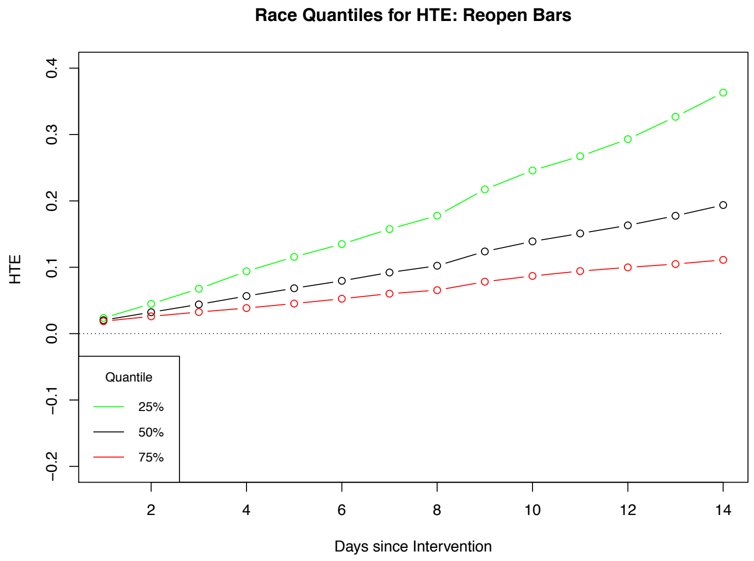

Figure S2: HTE of White for the NPI: Reopening bars. The effects are estimated with the moderator fixed at a given quantile and other variables fixed at the mean level.
